# Improved oral absorption and anti-lung cancer activity of paclitaxel-loaded mixed micelles

**DOI:** 10.1080/10717544.2016.1245370

**Published:** 2017-02-06

**Authors:** Jian Hou, E. Sun, Zhen-Hai Zhang, Jing Wang, Lei Yang, Li Cui, Zhong-Cheng Ke, Xiao-Bin Tan, Xiao-Bin Jia, Huixia Lv

**Affiliations:** 1 Affiliated Hospital of Integrated Traditional Chinese and Western Medicine, Nanjing University of Chinese Medicine, Nanjing, Jiangsu, China,; 2 Key Laboratory of New Drug Delivery System of Chinese Meteria Medica, Jiangsu Province Academy of Traditional Chinese Medicine, Nanjing Jiangsu, China,; 3 College of Pharmacy, Jiangsu University, Zhenjiang, Jiangsu, China, and; 4 College of Pharmacy, China Pharmaceutical University, Nanjing Jiangsu, China

**Keywords:** Paclitaxel, vitamin E-TPGS, Plasdone®S-630 Copovidone, micelles, oral bioavailability, Caco-2 monolayer, anti-tumor, gastrointestinal safety assay

## Abstract

The aim of this study was to establish a paclitaxel (PTX)-loaded mixed micelle delivery system (PTX-TP-M) with vitamin E-TPGS (TPGS) and Plasdone®S-630 Copovidone (PVPS630) as carriers to improve the solubility, oral absorption, and anti-tumor activity of PTX against lung cancer. In this study, PTX-TP-M was prepared using the ethanol thin-film dispersion method followed by characterization of the binary mixed micelles system. The average size of the PTX-TP-M was 83.5 ± 1.8 nm with a polydispersity index of 0.265 ± 0.007 and the drug loading (DL%) and entrapment efficiency (EE%) were 3.09 ± 0.09% and 95.67 ± 2.84%, respectively, which contributed to a high solubility of PTX about 24947-fold increase in water (4.78 ± 0.14 mg/mL). In addition, TEM analysis showed that the PTX-TP-M appeared spherical in structure and was well dispersed without aggregation and adhesion. *In vitro* release studies showed that the PTX-TP-M displayed a sustained release compared to free PTX in the dialysis bag. The efflux ratio of PTX reduced from 44.83 to 3.52 when formulated as PTX-TP-M; a 92.15% reduction, studied using the Caco-2 monolayer model. The oral bioavailability of PTX also improved by 4.35-fold, suggesting that PTX-TP-M can markedly promote the absorption in the gastrointestinal tract. Using *in vitro* MTT assays, it was observed that cytotoxicity was markedly increased, and IC_50_ values of PTX-TP-M (3.14 ± 0.85 and 8.28 ± 1.02 μg/mL) were lower than those of PTX solution (5.21 ± 0.93 and 14.53 ± 1.96 μg/mL) in A549 and Lewis cell, respectively. *In vivo* anti-tumor studies showed that PTX-TP-M achieved higher anti-tumor efficacy compared with PTX in Lewis bared C57BL/6 mice. Furthermore, a gastrointestinal safety assay also proved the safety of PTX-TP-M. All results demonstrated that the PTX-TP-M exhibited great potential for delivering PTX with increased solubility, oral bioavailability, and anti-cancer activity and this binary mixed micelles drug delivery system has potential to be used clinically.

## Introduction

Poor solubility in water has become one of the biggest challenges in pharmaceutical development, which greatly limits the emergence of commercial pharmaceutical products (George & Ghosh, 2013). Large amount of commercial active pharmaceutical ingredients (APIs) in development pipeline cannot be taken further in to the clinical arena due to poor aqueous solubility (Karwal et al., 2016). Although a variety of novel drug delivery system (DDS) technologies are being developed, such as micelles (Liang et al., 2015; Li et al., 2016), emulsions (Xiong et al., 2011; Liang et al., 2016), liposomes (Catalan-Latorre et al., 2016; Yang et al., 2016), and nanoparticles (Detappe et al., 2016) and other systems (Li et al., 2015), the feasibility of commercialization and scale up of these techniques are quite limited. Among them, self-assembled micelles are aggregates or a supramolecular assembly of surfactant molecules dispersed in a liquid colloid. This DDS forms a hydrophobic core that can entrap an insoluble drug; and the hydrophilic tails orient toward the aqueous solvent. This system can markedly enhance the solubility and bioavailability of insoluble drugs. Furthermore, some excipients used in the micelles can act as the P-glycoprotein (P-gp) inhibitors and promote intestinal absorption and inhibit efflux.

Paclitaxel (PTX), a broad-spectrum anti-cancer drug approved by FDA in 1992, has been used to treat ovarian, non-small cell lung, breast, and other cancers. Due to its poor aqueous solubility of about 0.19 μg/mL, absorption of PTX into blood is very low and does not reach the required concentration for activity (Bernabeu et al., 2016). The currently available commercial formulations are Abraxane®, Taxol®, Onxol®, and Nov-Onxol®, all of which are injections (Bernabeu et al., 2014), and may cause serious adverse reactions. A number of these side effects are associated with the excipients used, such as Cremophor EL, which contains polyoxyethylated castor oil. Dexamethasone is often given prior to beginning PTX treatment to mitigate some of the side effects. To overcome the above drawbacks, several approaches have been tried, including strategies for oral delivery. However, oral administration has challenges such as poor solubility, efflux from intestinal cells, low bioavailability, anti-cancer efficacy, and gastrointestinal tract irritation.

In this study, we chose two excipients vitamin E-TPGS (TPGS) and Plasdone®S-630 Copovidone (PVPS630) to form a binary mixed micelles system entrapping PTX. TPGS, comprising a hydrophilic polar head and lipophilic alkyl tail that contributes to a low critical micelle concentration (CMC) of 0.02% w/w and a HLB value of 13.2, is multifunctional drug carrier used in various delivery systems including prodrug-based strategies, micelles, liposomes, and nanoparticles (Zhang et al., 2012). As it formed by esterification of polyethylene glycol (PEG) and vitamin E, TPGS has the advantages of both. TPGS can be used as a self-assembling DDS to enhance solubility of poorly water-soluble drugs, extend half-life, enhance cellular uptake and to achieve sustained, controlled or targeted delivery, as well as to overcome multidrug resistance (MDR) by inhibiting P-gp-mediated efflux. Furthermore, the safety of TPGS has also been investigated; the acute oral median lethal dose (LD_50_) is more than 7 g/kg (TPGS/weight of rats) for young adult rats of both sexes. Owing to its versatility and safety, FDA has approved TPGS as a pharmaceutical excipient. PVPS630 is a random copolymer of N-vinyl-2-pyrrolidone and vinyl acetate. The addition of vinyl acetate to the hydrophilic vinyl pyrrolidone polymer chain enhances hydrophobicity, and hence PVPS630 is amphiphilic. In addition, PVPS630 is used as a tablet binder, matrix polymer for solid-dispersion formulations and film former for topical applications. It is commonly used to enhance the solubility of active insoluble drugs and to increase bioavailability through the formation of melt-extruded or spray-dried solid dispersions. In a study, PVPS630 was found to be the most effective among the polymers investigated and could form molecular dispersions up to 40% w/w drug loading (Zhao et al., 2012). In literature, PVPS630 has been used for preparing solid dispersions, not for the preparation of the micelles (Zheng et al., 2007,2008). In this study, we attempted to combine PVPS630 and TPGS to establish a binary mixed micelle system and achieved excellent water solubility, biocompatibility, and low toxicity.

In this study, we first investigated the solubilization capacity of this PTX-TP-M, followed by characterization of this binary mixed micelle system. The Caco-2 monolayer transport assay and oral bioavailability experiment were carried out to evaluate the oral absorption capability, along with and *in vivo* and *in vitro* evaluation of anti-tumor activity against lung cancer. Additionally, a gastrointestinal safety assay was performed to ensure the safety of this novel DDS.

## Materials and methods

### Materials

PTX (purity > 98%) was purchased from Melonepharma Technology Co., Ltd. (Dalian, China). Vitamin E-TPGS was purchased from Aladdin Industrial Co. Ltd. (Shanghai, China). PVPS630 was purchased from Sigma-Aldrich (St. Louis, MO). Cloned Caco-2 CT7 cells were provided by Dr. Moniqué Rousset of INSERM U178 (Villejust, France). A549 and Lewis lung cancer cells and basal Dulbecco’s modiﬁed Eagle’s medium (DMEM) were purchased from Nanjing KeyGEN Biotech. Co. Ltd. (Nanjing, China). Fetal bovine serum (FBS) was purchased from Gibco/BRL (Grand Island, NY). Milli-Q water (Millipore, Bedford, MA) was used for all experiments. Chromatographic grade methanol and acetonitrile were used (Tedia Company Inc., IA, USA). All other reagents were of analytical grade.

### Animals

Male Sprague-Dawley rats (200 ± 20 g) and male C57BL/6 mice (22 ± 2 g) were purchased from the SLAC Lab Animal Centre of Shanghai (Shanghai, China). All rats were given distilled water *ad libitum* and kept at a temperature of 25 °C and relative humidity of 45% for 2 weeks. All animal experiments were reviewed and approved by the Institutional Animal Care and Use Committee of the Jiangsu Provincial Academy of Chinese Medicine.

### Preparation of PTX-TP-M binary mixed micelles

PTX-TPGS-PVPS630 binary mixed micelles (PTX-TP-M) were prepared using the ethanol solvent evaporation method (Hou et al., 2016b). Briefly, 1000 mg TPGS, 500 mg PVPS630 and 50 mg PTX were dissolved in 10 mL ethanol, and the solution was vacuum-dried (−0.1 MPa) at a temperature of 45 °C to remove ethanol. The residue was rehydrated with 10 mL deionized water. The resultant suspension was then centrifuged at 16 000 rpm for 10 min to remove the un-entrapped drug and freeze dried.

### Characterization of PTX-loaded mixed micelles

The hydrodynamic diameter, polydispersity index (PDI) and zeta potential of the PTX-TP-M was measured using zetasizer (ZEN-3600, Malvern Instruments, Worcestershire, UK). The morphology of this binary micelle system was observed and photographed using transmission electron microscopy (TEM, JEM-200CX, JEOL, Japan).

The encapsulation efficiency (EE) and drug loading (DL) of drug in PTX-TP-M was calculated using the following formula (Hou et al., 2016a):

(1) $$\mathrm{Encapsulation}\;\mathrm{efficiency}\;(\%)\;=\;\frac{C_{\mathrm{PTX}\;\mathrm{remain}}}{C_{\mathrm{PTX}\;\mathrm{input}}}\;\times \;100\%$$

(2) $$\mathrm{Drug}\;\mathrm{loading}\;(\%)\;=\;\frac{C_{\mathrm{PTX}\;\mathrm{remain}}}{C_{\mathrm{TPGS}}\;+\;C_{\mathrm{PVPS}630}\;+\;C_{\mathrm{PTX}\;\mathrm{remain}}}\;\times \;100\%$$

where the *C*_PTX input_ is the concentration of PTX input before PTX-TP-M was formulated; *C*_PTX remain_ is the concentration of PTX after ultracentrifugation; *C*_TPGS_ and *C*_PVPS630_ are the concentration of TPGS and PVPS630 input. The concentration of PTX after ultracentrifugation was determined by HPLC instrument. The conditions for the UPLC method were as follows: Agilent HPLC instrument (Agilent 1260, Agilent Technologies, USA); column, Agilent Eclipse CDB-C18, 5 μm, 4.6 × 250 mm; mobile phase A was methanol, mobile phase B was acetonitrile, mobile phase C was water, A:B:C was 23:36:41; flow rate was 1.0 mL/min; column temperature was 30 °C, detection wavelength was 227 nm.

### *In vitro* drug-release profiles

The drug-release profiles of PTX-TP-M were obtained using the dialysis method. The formulations were put in dialysis membrane bags (MWCO 3500, Green Bird Inc., Shanghai, China) in PBS (300 mL, pH 7.4) and 0.5% (w/v) Tween 80 (Sinopharm Chemical Reagent Co., Ltd.) and stirred at 30 rpm, 37 °C for 48 h. At desired time intervals, 2 mL sample was withdrawn and replaced with an equal volume of the fresh release medium. For control samples, the same amount of PTX was dispersed in PBS and the release profiles were obtained in the same conditions. The amount of PTX was determined using HPLC described above.

### Cell culture

Three cell lines, namely Caco-2, A549, Lewis, were maintained in incomplete DMEM high-glucose medium supplemented with 10% FBS in an incubator (Thermo, MA, USA) at 37 °C, 95% relative humidity and 5% CO_2_. The cells were grown in 10 cm cell culture dishes to 80% confluency, and were detached from the plates using trypsin and transferred into Transwell® 6-well and 96-well plates respectively.

### Transport experiment

Caco-2 cells were used to study permeability of PTX (Jin et al., 2013b). At 21 days post-seeding in Transwell® 6-well plates, the Caco-2 cells form a well-developed monolayer with transepithelial electrical resistance (TEER) values greater than 350 Ω × cm^2^. To study permeability, PTX-TP-M loaded with 10 μM PTX was added to the apical (AP) or basolateral (BL) side and 400 μL of the donor samples and the receiver samples were collected at 0, 1, 2, 3, and 4 h, followed by replacement with the respective solutions (Jin et al., 2013a).The samples were then centrifuged at 16 000 rpm for 5 min at 4 °C. The supernatant containing PTX was measured using HPLC analysis described above. At the end of transport, TEER values were measured to ensure the integrity of the Caco-2 cell monolayers (Wan et al., 2015). The permeability of PTX was calculated using the following equation:

(3) $$p_{\mathrm{app}}\;=\;\frac{V}{S\times C}\;\times \;\frac{\mathrm{dC}}{\mathrm{dt}}\;=\;\frac{1}{S\times C}\;\times \;\frac{\mathrm{dM}}{\mathrm{dt}}$$

where *V* is the volume of the receiver (2.5 mL), *S* is the surface area of the cell monolayer (4.2 cm^2^), *C* is the initial concentration, *dC/dt* is the rate of concentration change in the receiver side, and *dM/dt* is the rate of drug transport. The rate of PTX transport was obtained by linear regression analysis.

### Pharmacokinetic evaluation in SD rats

For the pharmacokinetic studies, SD rats were randomly divided into two groups (*n* = 6). The PTX (suspended in CMC-Na solution) and PTX-TP-M were orally administered at a dose of 20 mg/kg to the two groups. Blood samples were collected to the heparinized tubes from the orbital vein at predetermined times (5, 15, 30, 60, 90, 120, 180, 240, 300, 360, 480, 720, 1440 min) and immediately centrifuged to obtain the plasma. Plasma (150 μL) was treated with 600 μL of methanol, and vortexed for 5 min to precipitate the plasma proteins. These samples were then centrifuged at 16 000 rpm for 10 min at 4 °C. After centrifugation, 20 μL of the samples were injected into the UPLC-MS/MS system for determination.

### Anti-tumor activity of PTX-loaded mixed micelles *in vivo* and *in vitro*

#### In vitro cytotoxicity

The *in vitro* anti-proliferative activity of PTX-TP-M was determined using MTT assay (Zhang et al., 2013; Najlah et al., 2016). A549 and Lewis cells were seeded in 96-well plates at a density of 1 × 10^5^ cells/well and incubated at 37 °C for 24 h. Fresh serum-free medium containing either PTX or PTX-TP-M was added to the wells. After 24 h, the medium was aspirated and the cells were incubated with 90 μL fresh medium and 10 μL MTT solution (5 mg/mL) for 4 h. The MTT formazan crystals were then dissolved in 100 μL DMSO solution and the plates was shaken for 10 min. The absorbance at 570 nm was measured using an iMark microplate reader (Bio-Rad Laboratories, USA). Each treatment was performed in triplicate. The inhibition of cell growth was calculated using the given equation [1 − (A_sample_−A_blank_/A_control_−A_blank_)] × 100%.

#### *In vivo* anti-tumor activity

C57BL/6 mice (22 ± 2 g) were maintained under pathogen-free conditions with normal access to food and water for 2 weeks. Lewis cells (1 × 10^6^) were suspended in serum-free DMEM (200 μL) and injected subcutaneously in the right flank of male mice. The mice were randomly divided into three groups (*n* = 6) after average tumor volume reached 50 mm^3^. Saline solution was administered to the control group, and PTX or PTX-TP-M was administered to the PTX group and the micelles group, respectively, at the same dose of 20 mg/kg. Mice were treated six times every 3 days and tumor volume and mice weight were measured on the day following administration. Tumor volumes were calculated based on the equation V= (length × width^2^)/2. At the end of the experiment, the animals were euthanised and the tumors were harvested for pathological analysis. Tumor tissues were immediately fixed in 10% formaldehyde-mixing fixative, and embedded in paraffin blocks after 24 h. The histologic tumor sections were placed on superfrost/Plus microscope slides. Hematoxylin and eosin (H&E) staining was used to evaluate the tumor pathological changes and the sections observed under a light microscope and photographed using a digital camera.

### Gastrointestinal safety studies

Gastrointestinal safety of the micelles was evaluated by H&E staining. Male SD rats were divided into two groups and treated orally with PTX or PTX-TP-M at a dose of 20 mg/kg every 2 days for 4 weeks. At the end of 4 weeks, the rats were euthanised, and the stomach and small intestine tissues were collected for pathological analysis as described in the section “In vivo anti-tumor activity”.

### Statistical analysis

The data were expressed as mean ± SD of independent experiments. One-way ANOVA was performed using SPSS 19.0 statistical software (SPSS Inc., Chicago, IL) to determine the difference among the samples. A value of *p* < 0.05 indicated a statistical significance compared to the control.

## Results and discussion

### Characterization of PTX-TP-M

**Figure 1.**
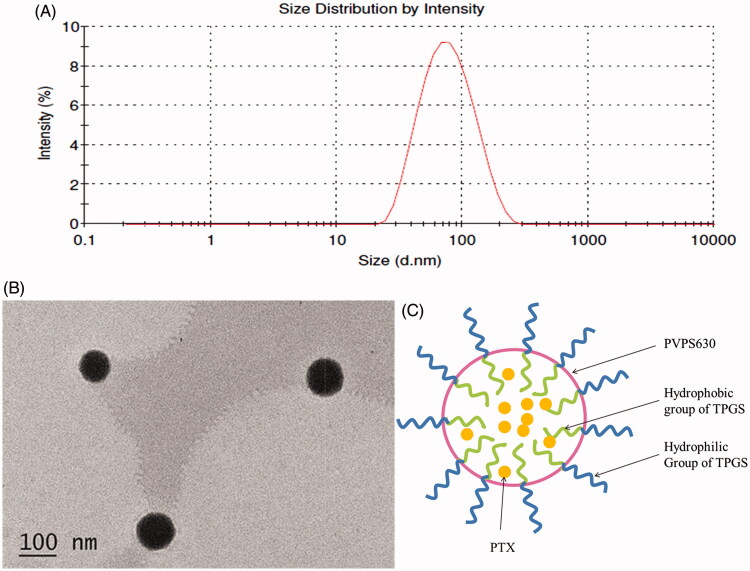
Size distribution of PTX-TP-M (panel A); TEM micrographs of PTX-TP-M (panel B); schematic illustration of the structure of PTX-TP-M (panel C). Scale bar = 100 nm.

Table 1 shows the characterization of self-assembly micelle system formed by TPGS and PVPS630. The PTX-TP-M solution was clear and transparent with average size of the micelles being 83.5 ± 1.8 nm and a PDI of 0.265 ± 0.007 (Figure 1A). Homogenous and small particle sizes could reduce the uptake by the reticuloendothelial system leading to passive accumulation in certain tissues and can directly affect the circulation time and bio-distribution *in vivo* of the carriers (Zhai et al., 2013; Zhu et al., 2014). Under TEM, the spherical morphology of PTX-TP-M was confirmed and there was no conglutination and adhesion (Figure 1B). As the theoretical CMC value for mixed surfactant system, 1/CMC*=X_1_/CMC_1_+ X_2_/CMC_2_ (X1, X2 and CMC_1_, CMC_2_ means the molar fractions and the CMC value of the expedient 1 and 2), the CMC value of the micelles formed by two excipients is lower than that of the single-excipient micelles. Micelles form when the concentrations of excipients are higher than the CMC value. The formation of the micelles can be understood using thermodynamics: micelles can form spontaneously because of a balance between entropy and enthalpy and the hydrophobic effects that forces the system to form a smaller energy system, which make the system more stable. So the stability of this binary mixed micelle system mixed was higher, and the micelles were easier to form. PVPS630 is a shell-like structure, when dissolved in water, the hydrophilic groups orient on the outside, while the hydrophobic group are facing inside. Based on the amphiphilic property of carrier material TPGS and PVPS630, insoluble drugs can be entrapped within the hydrophobic groups and form the core of this DDS, furthermore, hydrophilic group form the outer shell and reduce the interfacial tension of overall system in water (Figure 1C). This stable system can effectively improve system stability and drug solubility. Using HPLC analyses, drug loading (%) and encapsulation efficiency (%) were determined as 3.09 ± 0.09% and 95.67 ± 2.84%, respectively, which contribute to a high solubility of PTX reaching 4.78 ± 0.14 mg/mL. It can be concluded that most of the PTX was entrapped in the binary mixed micelle system.

**Table 1.** Characteristics of PTX-loaded mixed micelle system.

TPGS:PVPS630 (mg/mL:mg/mL)	Average size (nm)	PDI	DL%	EE%	Solubility (mg/mL)
100:50	83.5 ± 1.8	0.265 ± 0.007	3.09 ± 0.09	95.67 ± 2.84	4.78 ± 0.14

Data are presented as mean ± SD (*n* = 3).

### *In vitro* release

**Figure 2.**
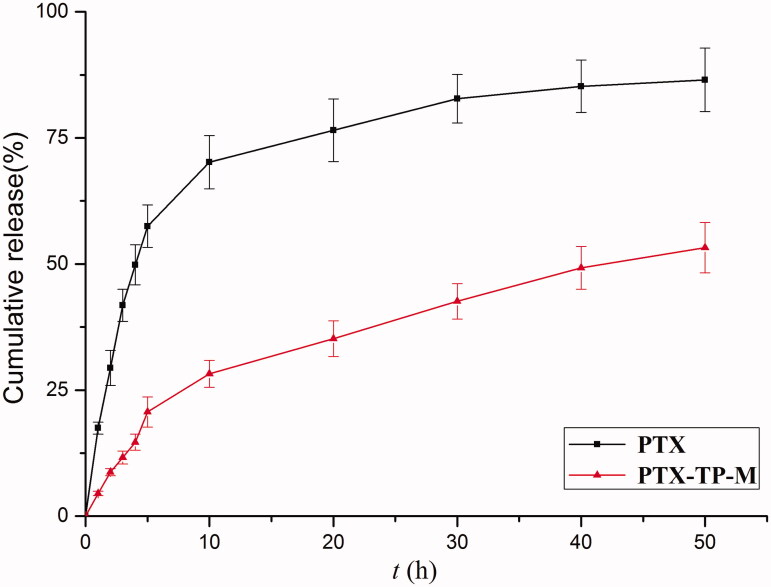
*In vitro* PTX release profiles from PTX-loaded mixed micelles and free drug at 37 °C over 48 h. Data are expressed as mean ± SD (*n* = 3).

For the *in vitro* release assays, the profile of PTX from free PTX and PTX-TP-M in simulated intestinal medium (PBS, pH 7.4) were shown in Figure 2. The results showed that PTX-TP-M had a slower release. For example, in the first 10 h, PTX showed a burst release of 70.2% while release from the PTX-TP-M micelles system was 28.2%. Furthermore, the PTX-loaded micelles sustained release over 48 h and the percentage of PTX released at 48 h was 53.2%, while the free drug was 86.2%. These results showed that release of PTX from the micelles was significantly sustained compared to free PTX. The slow drug-release from PTX-TP-M further confirmed the stability of this micelle system which can ensure that the PTX would remain encapsulated in inner core of micelles and achieve longer circulation times and slow release. The PTX might be delivered to the best absorption site slowly due to the release characteristics of PTX-TP-M in intestinal environment. Therefore, PTX-TP-M has a potential application in enhancing oral bioavailability of PTX and extending the residence time of PTX *in vivo* (Zhu et al., 2014).

### Transport experiment

**Figure 3.**
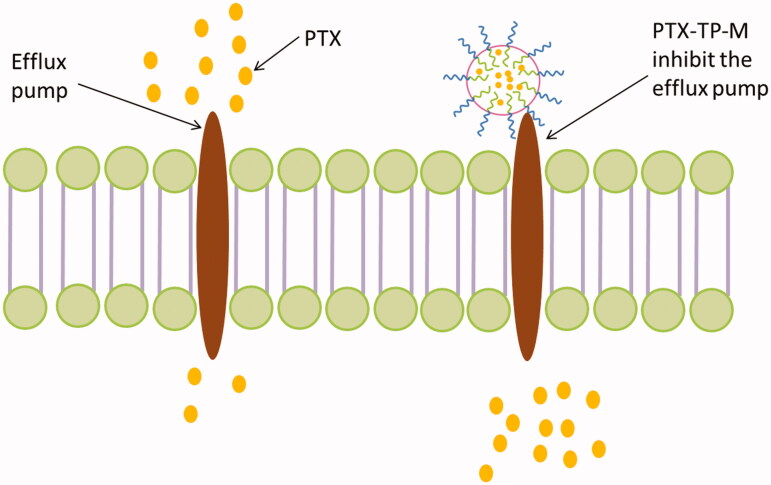
Schematic illustration of PTX-TP-M inhibits the efflux system and contributes to absorption.

In this study, the TEER values were measured before and after the experiment to ensure the excipients and drug in experimental concentration were nontoxic and the Caco-2 monolayer tight junctions were maintained (Moghimipour et al., 2016). As shown in Table 2, for transport of AP→BL, the *P*_appAB_ value of PTX-TP-M was 4.33 ± 0.41 × 10^−6^ cm/S, which was 3.71-fold higher than PTX (*P*_appAB_ = 0.92 ± 0.11 × 10^−6^ cm/S). This shows that the mixed micelle system composed of TPGS and PVPS630 significantly increased the absorption, which may be due to the enhancement of the solubility of PTX. In addition, both PVPS630 and TPGS alone can enhance the absorption of the drug, so their combination may play a synergistic effect in the absorption of PTX. The BL→AP transport aims to investigate the P-gp efflux inhibition of PTX-TP-M. The *P*_appBA_ of the PTX-TP-M group was 15.26 ± 1.58 × 10^−6^ cm/S which shows a significant decrease by 63.00% compared to the PTX group (41.24 ± 2.10 × 10^−6^ cm/S). This decrease is probably due to the addition of TPGS (Gao et al., 2016). TPGS acts as an effective efflux inhibitor, and can modulate efflux pumps such as P-gp or MDR-associated proteins. It often used as efflux inhibitor to address challenges associated with efflux-susceptible drugs (Assanhou et al., 2015). As PTX also has significant intestinal efflux phenomenon, we chose this biocompatible excipient as a part of novel DDS in our experiment, and effectively inhibited the efflux of PTX decreasing the efflux ratio from 44.83 to 3.52. These results indicate that the TPGS-PVPS630 binary mixed micelle systems could significantly increase the absorption and inhibit efflux of PTX (Figure 3).

**Table 2.** Permeability and efflux ratio of PTX and PTX-TP-M.

	*P*_app_ × 10^−6^ cm/s		
Group	AP-BL	BL-AP	Efflux ratio
PTX	0.92 ± 0.11	41.24 ± 2.10	44.83
PTX-TP-M	4.33 ± 0.41*	15.26 ± 1.58*	3.52

Absorption permeability was expressed as AP-BL, whereas secretory permeability was presented as BL-AP. Efflux ratio was *P*_app (BL-AP)_/*P*_app (AP-BL)_. Data expressed as mean ± SD (*n* = 3).

* Significant difference compared to PTX (*p* < 0.01).

### Pharmacokinetic evaluation in SD rats

**Figure 4.**
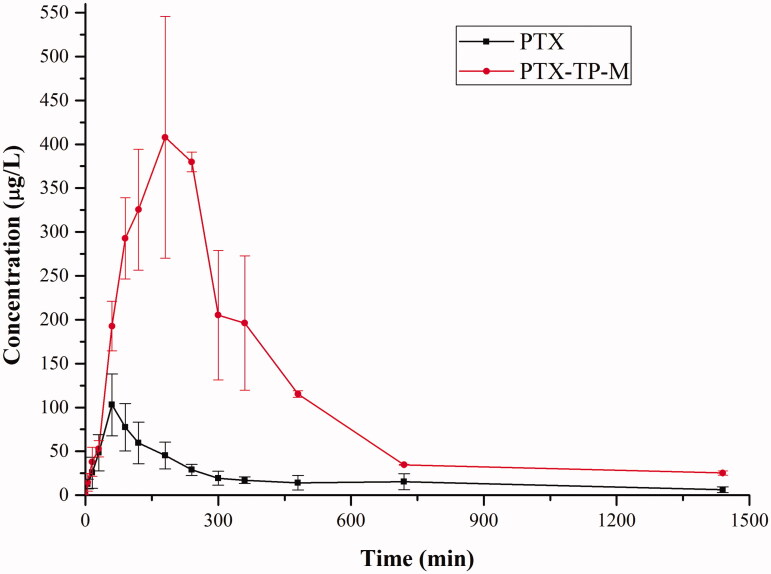
The plasma concentration-time curve of PTX in rats after oral administration of PTX, PTX-loaded mixed micelles (20 mg/kg, IS). Data are presented as mean ± SD (*n* = 6).

In this study, a single oral administration of 20 mg/kg of PTX and PTX-TP-M were given to SD rats in order to measure the concentration of PTX in plasma. Figure 4 and Table 3 show the plasma concentration-time curve and the pharmacokinetic parameters. The average value of *C*_max_ was 446.04 ± 76.75 μg/L with a *T*_max_ of 3.33 ± 0.58 h in PTX-TP-M group, while in the PTX group, the average value of *C*_max_ was 131.14 ± 23.55 μg/L after the oral administration of PTX with a *T*_max_ of about 1.08 ± 0.38 h. The higher *C*_max_ in PTX-TP-M group was probably due to the enhancement of solubility of PTX, and drug entrapped in micelles can obtain a sustained release. The average values of AUC_0–_*_t_* of PTX and PTX-TP-M in rats were 481.70 ± 84.18 and 2579.45 ± 306.32 μg/L × h, respectively. The micelles markedly improved the bioavailability of PTX, and it was more than five times that of the free drug. The increase of the relative bioavailability of the PTX-TP-M after oral administration could be attributed to the following reasons: (1) PTX solubility in water improved, and it was more readily absorbed in the intestine (Xu et al., 2015; Kim et al., 2016); (2) PVPS630 and TPGS can further promote the absorption of PTX (Ho et al., 2008); (3) TPGS inhibits the intestinal efflux pump (Wempe et al., 2009; Li et al., 2010). All these factors may have contributed to the increased bioavailability of PTX in the mixed micelle group.

**Table 3.** Pharmacokinetic parameters of PTX and PTX-TP-M (20 mg/kg, PTX).

Parameters	PTX	PTX-TP-M
AUC_0–_*_t_* (μg/L × h)	481.70 ± 84.18	2579.45 ± 306.32*
AUC_0–∞_ (μg/L × h)	676.08 ± 294.78	2640.68 ± 399.84*
MRT_0–_*_t_* (h)	6.94 ± 1.30	6.27 ± 0.41
MRT_0–∞_ (h)	14.46 ± 6.46	6.79 ± 0.55*
*t*_1/2_ (h)	10.21 ± 5.03	3.35 ± 1.72*
*T*_max_ (h)	1.08 ± 0.38	3.33 ± 0.58*
*C*_max_ (μg/L)	131.14 ± 23.55	446.04 ± 76.75*

Data are presented as mean ± SD (*n* = 6).

* Significant difference compared to PTX (*p* < 0.01).

### Anti-tumor activity

To determine anti-tumor efficacy of PTX-TP-M, two types of lung cancer cell lines including A549 and Lewis cells were evaluated by the MTT assay (Najlah et al., 2016). As shown in Table 4, the IC_50_ values of free PTX on A549 and Lewis cells were 5.21 ± 0.93 and 14.53 ± 1.96 μg/mL; whereas the IC_50_ values of PTX-TP-M decreased to 3.14 ± 0.85 and 8.28 ± 1.02 μg/mL, respectively in the two cell lines. It was thus observed that for lung cancer cells, PTX-TP-M exhibited a significantly higher cytotoxicity than PTX after 24 h of incubation (*p* < 0.01). The enhanced toxicity of this micelle system may be partially due to improved cellular uptake (Yang et al., 2016). Additionally, the presence of TPGS may contribute to the reduced IC_50_ as it itself possesses certain cytotoxicity (Lee et al., 2015).

**Table 4.** IC_50_ values against A549 and Lewis cells after 24-h treatment.

	IC_50_ (μg/mL)	
Cell line	PTX	PTX-TP-M
A459	5.21 ± 0.93	3.14 ± 0.85*
Lewis	14.53 ± 1.96	8.28 ± 1.02*

Data are presented as mean ± SD (*n* = 6). *Significant difference compared to PTX (*p* < 0.01).

**Figure 5.**
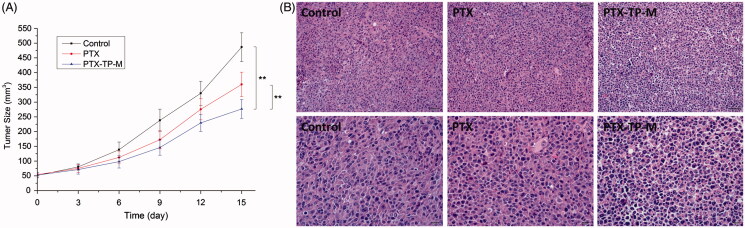
Tumor volumes of C57BL/6 mice implanted with Lewis cells in control, PTX, PTX-TP-M groups (panel A). The results are presented as the mean ± SD (*n* = 6); pathological section images of the tumor tissues of control and Lewis tumor-bearing C57BL/6 mice treated by PTX and PTX-TP-M (panel B). Scale bar = 20 or 50 μm.

In order to evaluate the anti-tumor activity of PTX-TP-M, the Lewis tumor model was established. The volume-time curve and images are shown in Figure 5(A). As seen in the Figure, from the 3rd day, two drug administration groups slightly showed an anti-tumor effect. Orally administered free PTX had some effect on the tumor, while the PTX-TP-M showed more significant effects. The percentage of tumor growth inhibition of PTX-TP-M was 43.13% at the 15th day, significantly higher than that of free PTX (26.01%). Figure 5(B) shows the H&E staining of tumor pathological changes. The control group shows that the tumor cells were mass volume large, densely arranged, and more mitotic, which represents less tumor necrosis, while in the PTX and PTX-TP-M group, after 15 days therapy, tumor cell volume had reduced, cells were arranged sparsely, and mitotic cells also reduced, and PTX-TP-M group showed better efficacy. All these results show that the mixed micelle system showed more effective suppression of tumor compared to the PTX group. The enhancement of anti-tumor activity contributes to the increasing of the absorption in gastrointestinal and the cellular in lung cancer cells.

### Gastrointestinal safety assay

**Figure 6.**
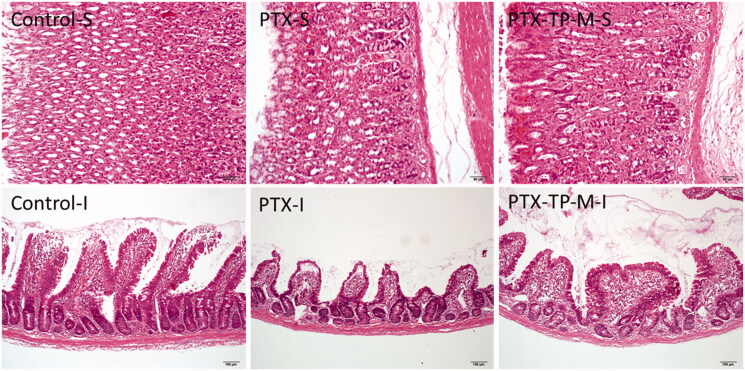
Gastrointestinal safety assay observed by H&E staining. (S) Stomach and (I) Intestine.

As shown in Figure 6, the administration of free PTX caused a slight degree of pathological change, and the micelles did not result in any further changes in the gastrointestinal system. In samples of the two administration groups, the surface of the gastric walls appeared slightly keratinized. In addition, the intestinal villi were shortened and relatively loose. These minor histological changes may due to the toxicity of PTX. Furthermore, there were no inflammatory cells found in two groups. All results demonstrated that the novel formulation did not increase the toxicity in gastrointestinal tract. This formulation is thus safe and can used in future application.

## Conclusion

A novel oral drug delivery micelle system formed by vitamin E-TPGS and PVPS630 nanoparticles was developed in this study for the purpose of enhancing the solubility and oral bioavailability of PTX. This system can entrap the drug in the core of the spherical micelles and sustain the release of PTX *in vitro*. The micelle system also significantly enhanced the permeability of PTX across the Caco-2 cell monolayer, and improved bioavailability of PTX in SD rats. The anti-cancer efficacy of PTX was significant improved *in vivo* and *in vitro*. In addition, the H&E staining confirmed gastrointestinal safety. Thus, this system has great potential for cancer therapy.
